# Supplementary material for: Draft Genome of Nocardia canadensis sp. nov. Isolated from Petroleum-Hydrocarbon-Contaminated Soil
Source: Microorganisms. 2023 Dec 12;11(12):2972. doi: 10.3390/microorganisms11122972 (PMC10745995; doi:10.3390/microorganisms11122972)
Supplement: Supplementary file 1 [file microorganisms-11-02972-s001.zip › microorganisms-2707857-supplementary.pdf]

**Supplementary Tables**

**Table S1** Genome Statistics of bacterial strain *Nocardia* sp. WB46.

| Attribute | Value |
|---|---|
| Genome size (bp) | 7,150,745 |
| Genes (total) | 6571 |
| CDSs[1] (total) | 6502 |
| Genes (coding) | 6387 |
| CDSs[1] (with protein) | 6387 |
| RNA genes | 69 |
| Complete rRNAs | 5 (5S) |
| Partial rRNAs | 5, 5 (16S, 23S) |
| tRNA genes | 51 |
| rRNA genes | 5, 5, 5 (5S, 16S, 23S) |
| ncRNAs | 3 |
| Pseudogenes | 117 |
| CDSs[1] (without protein) | 115 |
| Pseudogenes (frameshifted) | 37 of 117 |
| Pseudogenes (incomplete) | 82 of 115 |
| Pseudogenes (internal stop) | 13 of 115 |

[1] CDSs: Coding DNA Sequences

**Table S2** Scaffold (ordered contigs) organization of the genome of bacterial strain *Nocardia* sp. WB46.

| Contigs | Length (bp) | Avg. coverage | Notes |
|---|---|---|---|
| Contig_67 | 727310[1] | 112.71 | Contig_67 - (16S) |
| Contig_73 | 1130037 | 105.52 | (23S-5S) - Contig_73 - (16S) |
| Contig_5 | 72035 | 96.54 | (23S-5S) - Contig_5 - (16S) |
| Contig_26 | 542152 | 100.53 | (23S-5S) - Contig_26 - (16S) |
| Contig_3 | 235293 | 94.91 | (23S-5S) - Contig_3 - (16S) |
| Contig_100 | 99836 | 91.74 | (23S-5S) - Contig_100 - (GC-rich region) |
| Contig_60 | 131949 | 95.00 | (GC-rich region) - Contig_60 |
| Contig_90 | 2014676 | 90.74 | Contig_90 |
| Contig_11 | 90874 | 93.65 | Contig_11 |
| Contig_24 | 2106583 | 102.85 | Contig_24 Contig_67[†] |

[1] The scaffold is circular, with a 111 bp overlap between the end of Contig_24 and the beginning of Contig 67.

**Table S3** Genome sequencing of *Nocardia* canadensis strain WB46 has revealed genes linked to hydrocarbon degradation and plant growth-promotion traits.

| **Gene degradations** | **Candidate** | **Closest to** | **Similarity (%)** | **NCBI Reference Sequence (RefSeq)** |
|---|---|---|---|---|
| alkane 1-monooxygenase | pgaptmp_005297 | alkane 1-monooxygenase [*Rhodococcus ruber*] | 100.0 | WP_017682157.1 |
| | pgaptmp_001119 | alkane 1-monooxygenase [*Actinobacteria*] | 100.0 | WP_019048757.1 |
| | pgaptmp_001123 | alkane 1-monooxygenase [*Nocardia brasiliensis*] | 100.0 | WP_014987826.1 |
| Phosphate solubilization | pgaptmp_001057 | acid phosphatase [*Actinobacteria*] | 98.3 | WP_019048697.1 |
| Naphthalene dioxygenase | pgaptmp_004770 | aromatic ring-hydroxylating dioxygenase subunit alpha [*Frankia inefficax*] | 99.0 | WP_013424580.1 |
| | pgaptmp_004772 | aromatic ring-hydroxylating dioxygenase subunit alpha [*Gordonia rhizosphera*] | 98.9 | WP_006331860.1 |
| Siderophore utilization | pgaptmp_000075 | SIP domain-containing protein [*Nocardia* sp. MH4] | 97.8 | WP_218717909.1 |
| | pgaptmp_004292 | SIP domain-containing protein [*Nocardia* sp.] | 97.7 | WP_218719407.1 |
